# Identifying Candidate Genes Related to Soybean (*Glycine max*) Seed Coat Color via RNA-Seq and Coexpression Network Analysis

**DOI:** 10.3390/genes16010044

**Published:** 2025-01-01

**Authors:** Cheng Wang, Pingchun Fu, Tingting Sun, Yan Wang, Xueting Li, Shulin Lan, Hui Liu, Yongji Gou, Qiaoxia Shang, Weiyu Li

**Affiliations:** 1 College of Plant Science and Technology, Beijing Key Laboratory of New Agricultural Technology in Agriculture Application, National Demonstration Center for Experimental Plant Production Education, Beijing University of Agriculture, Beijing 102206, China; wangcheng1983@163.com (C.W.); 202330211015@bua.edu.cn (T.S.); lixueting@bua.edu.cn (X.L.); 13895838484@163.com (S.L.); 202220221092@bua.edu.cn (H.L.); 2 Key Laboratory for Northern Urban Agriculture of Ministry of Agriculture and Rural Affairs Beijing University of Agriculture, Beijing 102206, China; 202330221102@bua.edu.cn (P.F.); w1427783723@126.com (Y.W.); 18632230369@163.com (Y.G.)

**Keywords:** *Glycine max*, seed coat color, RNA-seq, candidate genes

## Abstract

Background: The quality of soybeans is reflected in the seed coat color, which indicates soybean quality and commercial value. Researchers have identified genes related to seed coat color in various plants. However, research on the regulation of genes related to seed coat color in soybeans is rare. Methods: In this study, four lines of seed coats with different colors (medium yellow 14, black, green, and brown) were selected from the F_2:5_ population, with Beinong 108 as the female parent and green bean as the male parent, and the dynamic changes in the anthocyanins in the seed coat were stained with 4-dimethylaminocinnamaldehyde (DMACA) during the grain maturation process (20 days from grain drum to seed harvest). Through RNA-seq of soybean lines with four different colored seed coats at 30 and 50 days after seeding, we can further understand the key pathways and gene regulation modules between soybean seed coats of different colors. Results: DMACA revealed that black seed coat soybeans produce anthocyanins first and have the deepest staining. Clustering and principal component analysis (PCA) of the RNA-seq data divided the eight samples into two groups, resulting in 16,456 DEGs, including 5359 TFs. GO and KEGG enrichment analyses revealed that the flavonoid biosynthesis, starch and sucrose metabolism, carotenoid biosynthesis, and circadian rhythm pathways were significantly enriched. We also conducted statistical and expression pattern analyses on the differentially expressed transcription factors. Based on weighted gene coexpression network analysis (WGCNA), we identified seven specific modules that were significantly related to the four soybean lines with different seed coat colors. The connectivity and functional annotation of genes within the modules were calculated, and 21 candidate genes related to soybean seed coat color were identified, including six transcription factor (TF) genes and three flavonoid pathway genes. Conclusions: These findings provide a theoretical basis for an in-depth understanding of the molecular mechanisms underlying differences in soybean seed coat color and provide new genetic resources.

## 1. Introduction

Soybean (*Glycine max*) seeds are rich in oil and protein and are widely used to extract vegetable oil and protein [1]. Soybean seeds contain eight essential amino acids that cannot be synthesized by the human body [2]. Seed coat color is an important morphological marker of soybean genetic evolution and an important attribute in determining the appearance of soybean seeds [3]. With the gradual evolution from wild soybeans to cultivated soybeans, seed coat colors have gradually increased, such as seeds colored yellow, green, brown, black, and two colors [4]. The color of the seeds affects their chemical composition. Compared with most yellow seeds, black and brown seeds usually contain more anthocyanins in the seed coat, and their antioxidant properties and edible flavor have obvious advantages [5]. The contents of soluble protein, fat, and mineral components (calcium, phosphorus, etc.) in green seeds are greater than those in yellow seeds, and the contents of dietary fiber and galactose in yellow seeds are significantly greater than those in green soybeans [6]. Pigmentation of the soybean seed coat affects the content of isoflavones and the composition and content of fatty acids [5,6].

Seed coat color is also an evolutionary characteristic of the subgenus Soybean [3]. During the domestication process, cultivated soybeans of various colors evolved from wild black soybeans. The uneven distribution of pigmentation can affect the appearance of soybean seeds and thus reduce their commercial value [7]. All glycine-containing soybean accessions in the USDA GRIN germplasm collection have black seed coats, but most soybean accessions have yellow seed coats. Although there is a small market for black soybeans, owing to the importance of light-colored varieties to produce natto and tofu, all modern high-yielding varieties are almost exclusively yellow-coated soybeans, with occasional occurrences of a range of other colors (brown, black, not quite black, light yellow, and yellow) [8]. Seed coat and hilum color are relatively simple epistatic polygenic traits. In the hilum and seed coat, pigmentation results from the interaction of four independent loci: Inhibitor (I), Tawny (T), the unnamed locus of R, and the flower color locus W1. However, the functions of these loci have not been annotated, and their genetic functions are still unclear [9,10,11,12,13]. The black seed coat trait is regulated mainly by the metabolic pathways of two anthocyanins: anthocyanin-3-O-glucoside and delphinidin-3-O-glucoside. In mature soybeans with brown seed coats, only anthocyanins are detected [14].

In recent years, with the continuous development, optimization, and large-scale application of sequencing technology, increasing evidence has shown that the regulation of RNA transcription plays an important role in plant growth and development [15,16]. Transcriptome sequencing (RNA-seq), one of the most commonly used second-generation high-throughput sequencing methods, has been widely used in the study of plant growth and development [15,16,17]. Through integrated analysis of RNA-seq data from four soybean lines at seven developmental stages, four molecular modules strongly related to isoflavone accumulation were obtained [18]. Through RNA-seq of 21 samples of B73 corn from 5 days before fertilization to 32 days after fertilization, a total of 25,346 genes involved in pericarp development were detected, 598 pericarp-specific genes were identified, and the key mechanisms and regulatory networks involved in corn pericarp development were elucidated [19]. Through spatiotemporal RNA-seq analysis of endosperm and embryonic tissues of *Vicia faba* at different developmental stages, the gene expression patterns, transcription networks, and core pathways of *V. faba* were revealed, and *LEC1*, *LEC2*, *FUS3*, and *ABI3* were identified as key players in the network [20]. RNA-seq of two peanut varieties with pink and purple seeds revealed 14 candidate genes related to anthocyanin biosynthesis, including a new gene, *Ah21440*, related to hydroxycinnamyl transferase (HCT) biosynthesis [21]. Recently, genetic methods such as bulked segregant analysis RNA-seq (BSR-Seq), map-based cloning, and transgenic technology have been used to successfully identify the key factor that regulates soybean pod skin color: the *L1* gene (*Glyma.19g120400*). *L1* can catalyze the Claisen condensation-like reaction between acetyl-CoA and 4-hydroxyphenylpyruvate (4-HPP) to generate eucomic and piscidic acids. Soybean seed coat color is a basic biological trait and an important agronomic trait that describes a variety of characteristics [22]. Researchers have identified genes related to the color of seeds, flowers, and other organs of various plants through omics data and constructed a gene regulation network. However, few studies have investigated genes that regulate seed coat color in soybeans. Therefore, in this study, RNA-seq was performed on soybean kernels with black, brown, and green seed coats and yellow seed coats produced by the F2:5 segregation population with a green seed parent coat color (Beinong 108 × green beans) at 30 and 50 days of age. The RNA-seq data were subjected to cluster analysis, difference analysis, GO and KEGG enrichment analysis, and TF expression analysis. By constructing a weighted gene coexpression network, candidate genes and regulatory pathways related to soybean seed coat color were identified. This study provides a theoretical basis for an in-depth understanding of the molecular mechanism of differences in the color of soybean seed coats and provides new genetic resources.

## 2. Materials and Methods

### 2.1. Plant Material

**Figure 1:**
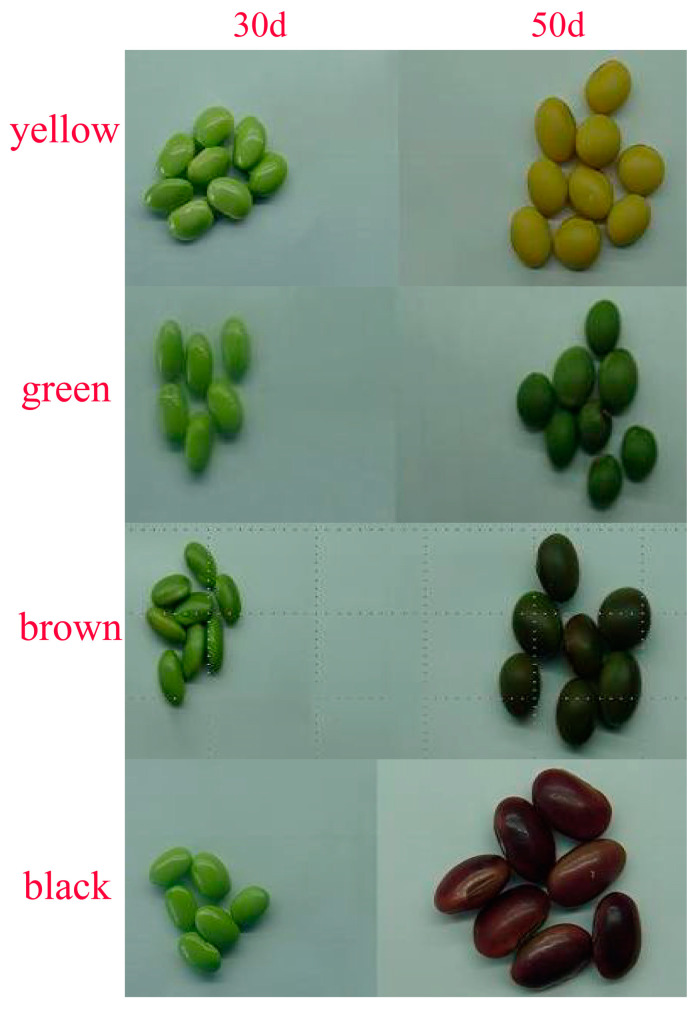
Four different soybean seed coat colors after 30 days and 50 days of drumming.

In this study, the soybean strain Beinong 108 was used as the female parent, and the green bean was used as the male parent. F_1_ was obtained after crossbreeding. The F_1_ generation from the cross was then continuously selfed for 4 years to obtain an F_2:5_ segregating population of 1139 lines. The seed coat colors of the parents Beinong 108 and lima bean are green, and the offspring have three seed coat colors: green, black, and brown (Figure S1). The test lines were planted in the eastern area of Beijing Agricultural College on 10 June 2019. The green seed coat, black seed coat, and brown seed coat were separated from the yellow seed coat (Zhonghuang 14) and (Beinong 108 × green bean) F_2:5_. The soybean seed coats 30 days after seeding (when the color of the seed coat had not changed) and 50 days after seeding (when the color of the seed coat had completely changed) were quickly frozen in liquid nitrogen in 1.5 µL centrifuge tubes and stored in an ultralow-temperature refrigerator at −80 °C (Figure 1).

### 2.2. Fixation and Staining

Sampling and dyeing were carried out every 3 days after 20 days of drumming and every 2 days after 44 days of drumming until the seeds were harvested. The sampling parts were all pods in the top second segment (to ensure three biological replicates for each variety). The entire pod was removed during sampling, and the seed coat was peeled off for subsequent tests. The soybean seeds were placed in the prepared AF fixative (85 mL of 95% ethanol, 10 mL of formaldehyde (concentrated), and 5 mL of glacial acetic acid added to 100 mL of the solution) and fixed at 6 °C for more than 24 h. The fixed seeds were removed, and the seed coat was peeled off for dyeing. The seed coat was placed into a prepared acid–alcohol solution of 4-dimethylaminocinnamaldehyde (DMACA) (0.5 g DMACA in 100 mL, 50–55% ethanol, and 10–15% hydrochloric acid). The DMACA powder was orange, the DMACA acidic–alcohol solution was orange-yellow, and images were taken after 2 h of dyeing.

### 2.3. RNA-Seq Sequencing

RNA was extracted via the TRIzol method, and RNA integrity was detected via 1% agarose gel electrophoresis. The RNA was stored in a −80 °C freezer, after which the extracted total RNA was transferred to Beijing Histology Biotechnology Co., Ltd. (Beijing, China) for sequencing on dry ice. The extracted RNA was fragmented via a PCR plate with a magnetic plate holder. Fragmented mRNA was reverse transcribed into cDNA via Superscript II and random primers (Invitrogen, Carlsbad, CA, USA). The RNA-seq library preparations were sequenced on an Illumina HiSeq 2500/X platform, and 150 bp paired-end reads were generated. Data filtering and quality control were performed through the fastp software (version 0.23.4), and the resulting clean data were used for subsequent analysis [23]. The soybean genome (http://plants.ensembl.org/Glycine_max/Info/Index, (accessed on 8 March 2024)) was used as a reference, and TopHat2 was used for read comparison [24]. The Cuffquant and Cuffnorm components of the Cufflinks software (version 2.2.1)were used to quantify the expression levels of transcripts through the position information of mapped reads on genes [25]. Gene expression was measured via the fragments per kilobase of transcript per million mapped reads (FPKM) method.

### 2.4. Identification of Differentially Expressed Genes

EBSeq was used to perform differential expression analysis to calculate the differential expression folds of genes between different samples on the basis of their expression levels [26]. The absolute value of FDR ≤ 0.01 and log2-fold change ≥ 1 were used as the standards to screen for differentially expressed genes (DEGs). Gene Ontology (GO) enrichment analysis of DEGs was performed via the GOseq R package, in which gene length bias was corrected [27]. GO terms with corrected *p* values less than 0.05 were considered significantly enriched with DEGs. The amino acid sequences of all the DEGs were submitted to the KEGG (https://www.genome.jp/kegg/, (accessed on 15 April 2024)) database for pathway annotation. Transcription factor prediction uses the iTAK (http://itak.feilab.net/cgi-bin/itak/index.cgi, (accessed on 22 April 2024)), integrates and optimizes the PlnTFDB and PlantTFDB databases, uses the TF (transcription factor) families and rules defined in the database, and identifies TFs through BLASTX [28].

### 2.5. Construction of the Coexpression Network

The R language WGCNA software (version 1.73) package was used to perform coexpression analysis on the gene expression profiles of the DEGs through the dynamic branch-cutting method [29]. To ensure the distribution of the scale-free network, the weighting coefficient β should satisfy the correlation coefficient close to 0.8 and have a certain degree of gene connectivity. In this work, β = 7 is chosen as the weighting coefficient. The automatic network construction function of blockwise modules was used to construct the network. Multiple valid modules were obtained, and the number of genes contained in each module differed. Using minModuleSize = 30 and Merge Cut Height = 0.25 as standards, modules with a similarity of 0.75 were merged. The correlation coefficient between the module’s characteristic vector ME (module eigengene) and different lines was calculated. The coexpression network was visualized via the Cytoscape (version 3.10.1) software [30].

### 2.6. Quantitative Real-Time PCR

Total RNA was extracted via the RNAprep Pure Polysaccharide and Polyphenol Plant Total RNA Extraction Kit (Tiangen, Beijing, China). The concentration of each RNA sample was determined via a NanoDrop 2000 spectrophotometer (Thermo Fisher Scientific, Waltham, MA, USA), and 1 μg of isolated RNA was subsequently used to obtain first-strand cDNA via reverse transcription with a PrimeScript™ RT Kit with gDNA Eraser (Takara Bio Inc., Shiga, Japan). qRT–PCR analysis was performed using Roche LC480 equipment (Roche Diagnostics GmbH, Mannheim, Germany) and SYBR Green (Takara Bio, Inc.). A two-step PCR amplification procedure was used, with predenaturation at 95 °C for 30 s, followed by 40 cycles of denaturation at 95 °C for 5 s, and annealing at 60 °C for 34 s. Amplification, dissolution, and standard curves were automatically generated by the Roche LC480 software. The results were analyzed relatively quantitatively via the 2^−ΔΔCt^ method. The internal reference gene was ACTIN, and each procedure involved three biological replicates [31]. All primers used in this study are listed in Table S1.

## 3. Results

### 3.1. Distribution of Anthocyanins in Different Soybean Seed Coat Colors

**Figure 2:**
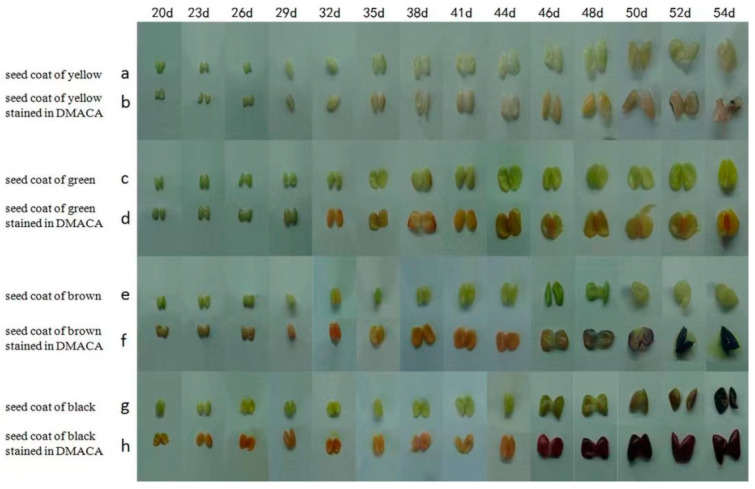
DMACA staining images of the seed coat bulging into four colors from 20 d to 54 d. (**a**) The yellow seed coat is unstained, (**b**) the yellow seed coat is stained, (**c**) the green seed coat is unstained, (**d**) the green seed coat is stained, (**e**) the brown seed coat is unstained, (**f**) the brown seed coat is stained, (**g**) the black seed coat is unstained, and (**h**) the black seed coat is stained.

DMACA can combine with proanthocyanidins in plant tissues or organs in acidic alcohol solutions to dye the plant tissues or organs blue [32]. To evaluate the anthocyanin content of the four different soybean seed coat colors, samples were taken every 3 days after 20 days of seeding and every 2 days after 44 days of seeding until seed harvest (Figure 2). The color of the yellow seed coat did not change during dyeing, but the hilum and surrounding parts were dyed blue by the DMACA staining solution 50 days after seeding (Figure 2a,b). The green-colored seed coats of the soybeans did not change during the dyeing test (Figure 2c,d). The brown seed coat of soybeans began to be dyed blue by the dye 48 days after seeding and was completely dyed blue 52 days after seeding, which shows that brown soybeans begin to produce proanthocyanidins 48 days after seeding, which gradually increase over time (Figure 2e,f). The black seed coats of the soybeans began to change 46 days after being drummed, and the black seed coat is stained blue-purple by the dye. This shows that black soybeans produce the most anthocyanins and are produced earliest (Figure 2g,h). To identify the regulatory network and key genes involved in soybean seed coat color, RNA-seq was performed on the soybean seed coats of these four lines 30 days after seeding (the seed coat did not change color) and 50 days after seeding (the seed coat completely changed color).

### 3.2. Overall Analysis of RNA-Seq Data

After RNA-seq filtering of eight samples of the four soybean lines tested in this study, 57.48 Gb of effective bases were obtained. The number of effective bases obtained from each sample reached at least 5.81 Gb, the Q20 percentage ranged from 97.45–98.17%, with an average of 97.74%, and the Q30 percentage ranged from 92.97–94.49%, with an average of 93.57%. The average alignment rate with the reference genome was 91.11%; therefore, transcriptome sequencing produced high-quality data (Table S2). We conducted annotations from the COG, GO, KEGG, KOG, Pfam, SwissProt, TrEMBL, and nr databases (Table 1). A total of 56,848 transcripts were annotated from eight databases, including 17,860 COG, 42,447 GO, 11,047 KEGG, 28,613 KOG, 43,237 Pfam, 42,272 SwissProt, 55,817 TrEMBL, and 26,835 Nr. The annotation information of these genes provides a solid basis for further analysis of transcript changes among the four soybean seed coats.

**Figure 3:**
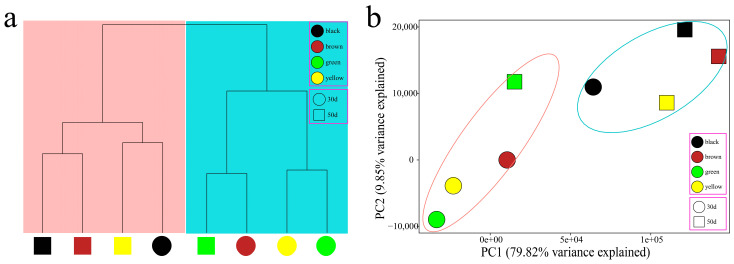
(**a**) Cluster dendrogram showing the two different developmental stages of the dynamics of changes in the color of the soybean seed coat. (**b**) PCA of eight RNA-seq samples.

To further analyze the transcriptome dynamics of soybean seed coat color changes, we performed PCA and hierarchical clustering on samples from four lines at two developmental time points (Figure 3a,b). The RNA-seq samples were divided into two groups. The green, yellow, and brown samples at the earliest time point (30 days) and the green samples at 50 days formed the first group, which represented the development of the green stage of the soybean seed coat. The 30-day black and 50-day yellow, brown, and black samples formed the second group, representing the beginning of anthocyanin synthesis in soybean seed coat development. Interestingly, the black sample was in the second group at 30 days, and the tests of the black sample were also the first to synthesize anthocyanins. This shows that the main reason for the difference in soybean seed coat color is the difference in development at 30–50 days after seeding, which is consistent with the results of DMACA staining.

### 3.3. Difference Analysis

**Figure 4:**
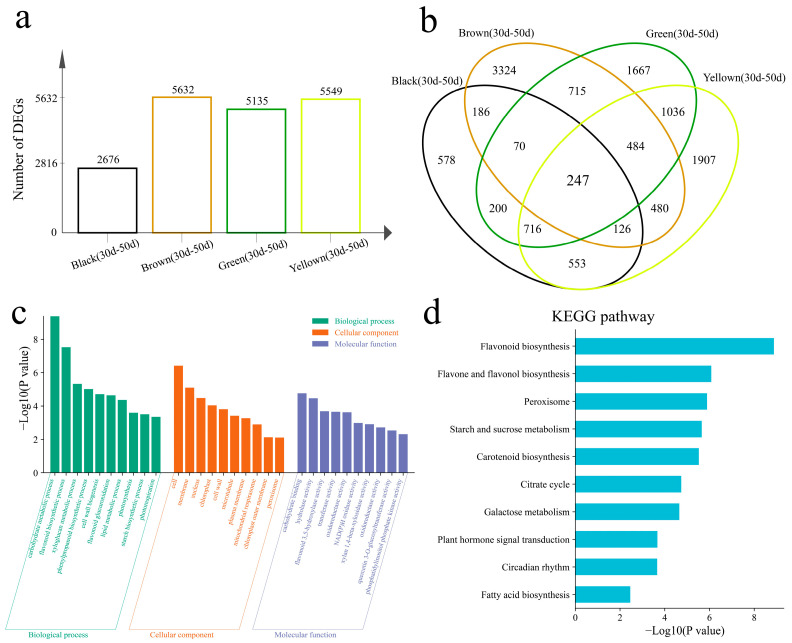
(**a**) Number of differentially expressed genes in different lines. (**b**) Venn diagram of differentially expressed genes within different lines. (**c**) GO enrichment analysis of differentially expressed genes within the lines. (**d**) KEGG enrichment analysis of differentially expressed genes within the lines.

Differential expression analysis of the same line at different developmental stages revealed 2676 DEGs in black, 5632 DEGs in brown, 5135 DEGs in green, and 5549 DEGs in yellow (Figure 4a). The number of DEGs in black is the smallest, which also shows that the differences between black samples are small, which is consistent with the results of PCA and hierarchical clustering. There were 247 common DEGs in the four lines, and 578 DEGs were uniquely differentially expressed in the black line, 3324 DEGs were uniquely differentially expressed in the brown line, 1667 DEGs were uniquely differentially expressed in the green line, and 1907 DEGs were uniquely differentially expressed in the yellow line (Figure 4b). To elucidate the functions of all 11,792 DEGs, GO enrichment analysis was performed on all DEGs within the lines (Figure 4c). The biological processes annotated were the carbohydrate metabolic process, flavonoid biosynthetic process, xyloglucan metabolic process, phenylpropanoid biosynthetic process, cell wall biogenesis, photosynthesis, and photorespiration. The KEGG pathways of all DEGs between the lines were the flavonoid biosynthesis, flavone and flavonol biosynthesis, peroxisome, starch and sucrose metabolism, carotenoid biosynthesis, citrate cycle, galactose metabolism, plant hormone signal transduction, circadian rhythm, and fatty acid biosynthesis pathways (Figure 4d).

**Figure 5:**
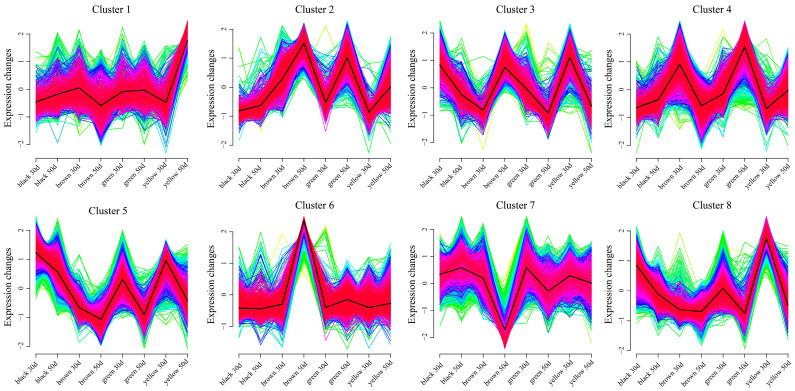
Line chart of DEG expression patterns from different developmental periods (30 d vs. 50 d) of the same lines.

Using the k-means clustering method, a total of eight statistically significant clusters were identified from 11,792 DEGs in the lines (Figure 5). Cluster 1 presented the highest expression level in the yellow line at 50 days, and its expression level in the other lines remained unchanged. The expression level of Cluster 2 increased with development in the four lines, and the expression level was the highest at 50 days in the brown and green lines. In Cluster 3, except for the increase in expression in brown, the expression in the other three lines decreased. The change trend of Cluster 4 is opposite to that of Cluster 3. The expression in brown decreased, whereas the expression in the other three lines increased. The expression level of Cluster 5 decreased in all the lines, and the expression level was highest in the black line at 30 days. The expression level of Cluster 6 in the brown line increased, and the expression level was the highest at 50 days. The expression levels in the other lines did not change. The expression level of Cluster 7 in the brown line decreased, and the expression level was the lowest at 50 days. The expression levels in the other lines did not change. The expression level of Cluster 8 decreased in all the lines, and the expression level of the yellow line was the highest at 30 days.

**Figure 6:**
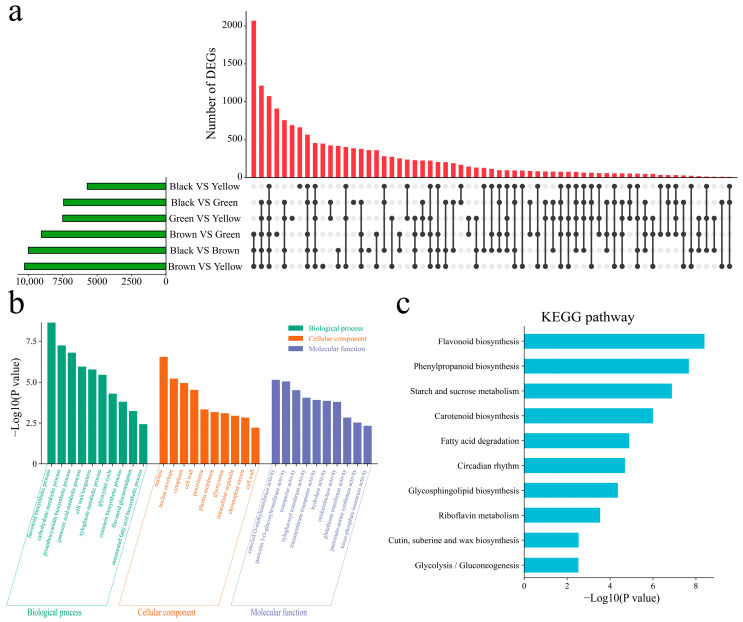
(**a**) UpSet plots of differentially expressed genes from the same developmental period of different lines. (**b**) GO enrichment analysis of DEGs in the same developmental period of different lines. (**c**) KEGG enrichment analysis of DEGs in the same developmental period of different lines.

DEGs were also identified between different lines. There were 9996 DEGs between black and brown plants, 7436 DEGs between black and green plants, 5722 DEGs between black and yellow plants, and 9051 DEGs between brown and green plants and between brown and yellow plants. There were 10,274 DEGs between the green and yellow varieties and 7051 DEGs between the green and yellow varieties (Figure 6a). There were 1073 common DEGs among the four lines; 363 DEGs were uniquely differentially expressed between black and brown, 378 DEGs were uniquely differentially expressed between black and green, 662 DEGs were uniquely differentially expressed between black and yellow, 910 DEGs were uniquely differentially expressed between brown and green, 447 DEGs were uniquely differentially expressed between brown and yellow, and 691 DEGs were uniquely differentially expressed between green and yellow. To elucidate the functions of all 15,651 DEGs between the lines, GO enrichment analysis was performed on all DEGs within the lines (Figure 6b). The main annotations were the flavonoid biosynthetic process, carbohydrate metabolic process, proanthocyanidin biosynthetic process, jasmonic acid metabolic process, cell wall biogenesis, xyloglucan metabolic process, glyoxylate cycle, coumarin biosynthetic process, and flavonoid glucuronidation. The KEGG pathways associated with the DEGs between the lines included flavonoid biosynthesis, phenylpropanoid biosynthesis, starch and sucrose metabolism, carotenoid biosynthesis, fatty acid degradation, circadian rhythm, glycosphingolipid biosynthesis, and riboflavin metabolism (Figure 6c).

**Figure 7:**
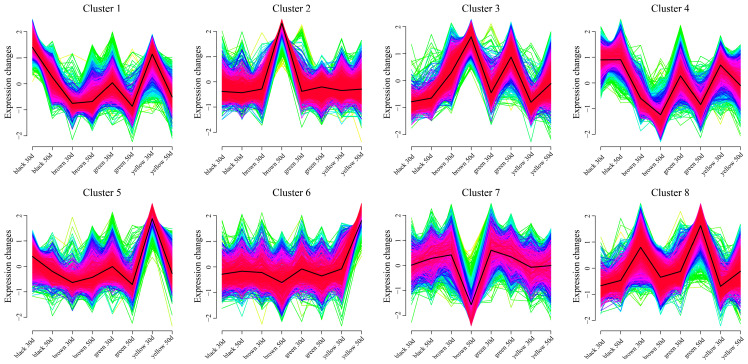
Line chart of DEG expression patterns for the same developmental period of different lines.

Using the k-means clustering method, eight statistically significant clusters were identified among 15,651 DEGs between different lines (Figure 7). The expression level of Cluster 1 increased slightly along the brown line, whereas the expression level decreased in the other lines. The expression level was the highest in the black and yellow lines at 30 days. The expression level of Cluster 2 in the brown line increased for 50 days and reached the maximum value, whereas the expression level in the other lines remained unchanged. The expression level of Cluster 3 increased in all the lines, and the expression level was the highest at 50 d of the brown line. The expression level of Cluster 4 genes in black essentially remained unchanged, whereas the expression levels in the other three lines decreased. The expression level of Cluster 5 in the brown line decreased for 30 days and was the highest, whereas the expression level in the other lines remained unchanged. The expression level of Cluster 6 in the yellow line increased, and the expression level was the highest at 50 days. The expression levels in the other lines did not change. The expression level of Cluster 7 in the brown line decreased, and the expression level was the lowest at 50 days. The expression levels in the other lines did not change. The expression level of Cluster 8 decreased in the brown line and increased in the other lines. The expression level of the green line was the highest at 50 d.

### 3.4. Analysis of Differentially Expressed Transcription Factors

**Figure 8:**
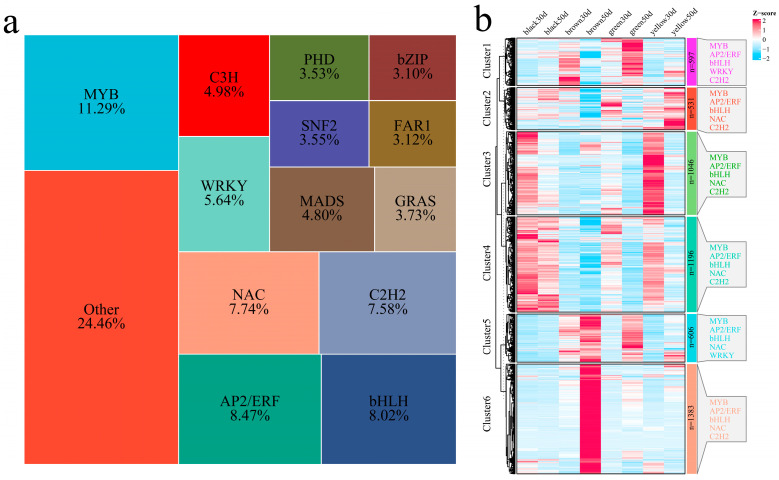
(**a**) Treemap of the percentage of TFs within the line. (**b**) Clustering heatmap of TFs within the line. The right side shows the number of TFs in the top five clusters.

A total of 5359 TFs were identified among the 16,456 DEGs from different developmental periods of the same lines and the same developmental period of different lines, including mainly MYB, AP2/ERF, bHLH, NAC, C2H2, WRKY, C3H, MADS, GRAS, SNF2, PHD, FAR1, and bZIP (Figure 8a). The differentially expressed transcription factors were divided into six main clusters with different expression patterns through hierarchical clustering (Figure 8b). Cluster 1 included 597 TFs, with the highest expression levels in the brown 30 d and green 50 d groups, which included mainly MYB, AP2/ERF, bHLH, WRKY, and C2H2. Cluster 2 included 531 TFs, with the highest expression levels occurring at 50 d (green) and 50 d (yellow), which were mainly MYB, AP2/ERF, bHLH, NAC, and C2H2. Cluster 3 included 1046 TFs, with the highest expression levels in the black 30 d and yellow 30 d groups, which included mainly MYB, AP2/ERF, bHLH, NAC, and C2H2. Cluster 4 included 1196 TFs, with the highest expression levels in the 30 d and 50 d black plants; these genes included mainly MYB, AP2/ERF, bHLH, NAC, and C2H2. Cluster 5 included 606 TFs, with the highest expression levels in the brown 50 d and green 50 d groups, which included mainly MYB, AP2/ERF, bHLH, NAC, and WRKY genes. Cluster 6 included 1383 TFs, with the highest expression level in the brown 50 d group, mainly the iMYB, AP2/ERF, bHLH, NAC, and C2H2 genes.

### 3.5. Weighted Gene Coexpression Network Construction and Candidate Gene Mining

**Figure 9:**
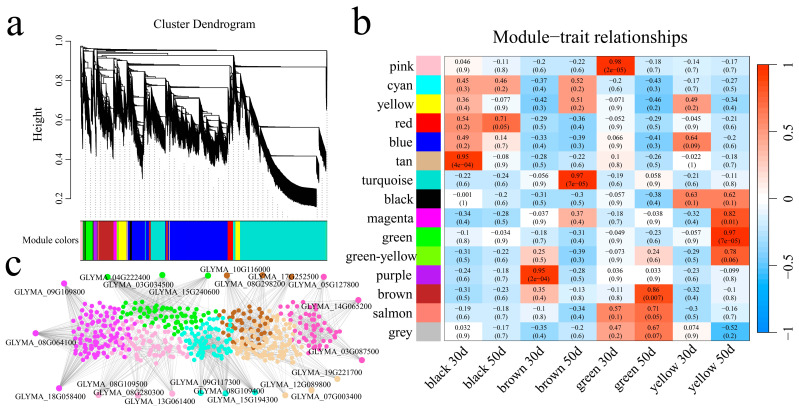
(**a**) Hierarchical clustering tree for gene coexpression network analysis. (**b**) Heatmap of correlations and significance between modules and different samples. (**c**) Gene coexpression network module; different colors were used to represent different modules.

Weighted gene coexpression network analysis (WGCNA) was used to construct a coexpression network of 16,456 DEGs from different developmental periods of the same lines and the same developmental period of different lines related to soybean seed coat color (Table S3). The dynamic shear tree method was used to merge modules with similar expressions, and 15 coexpression modules were obtained, with different colors used to represent different modules (Figure 9a). According to the correlation results between modules and lines, the pink module was strongly correlated with the green color at 30 d, the tan module was strongly correlated with the black color at 30 d, the turquoise module was strongly correlated with the brown color at 50 d, the magenta and green modules were strongly correlated with the yellow color at 50 d, the purple module was strongly correlated with the brown color at 30 d, and the brown module was significantly highly correlated with the green color at 50 d (Figure 9b). Pink, tan, turquoise, magenta, green, purple, and brown were selected to construct the gene interaction network and identify the core genes (Figure 9c and Table S4). Three core genes were identified for each module, resulting in 21 core genes. These 21 core genes included six TFs (*GLYMA_08G280300* (AP2/ERF), *GLYMA_07G003400* (bHLH), *GLYMA_19G221700* (WRKY), *GLYMA_03G087500* (MYB), *GLYMA_09G109800* (MADS), and *GLYMA_08G298200* (MYB)), and three flavonoid pathway genes (*GLYMA_08G109500* (CHS), *GLYMA_08G109400* (CHS), and *GLYMA_04G222400* (CHI)) (Table 2).

### 3.6. qRT–PCR

**Figure 10:**
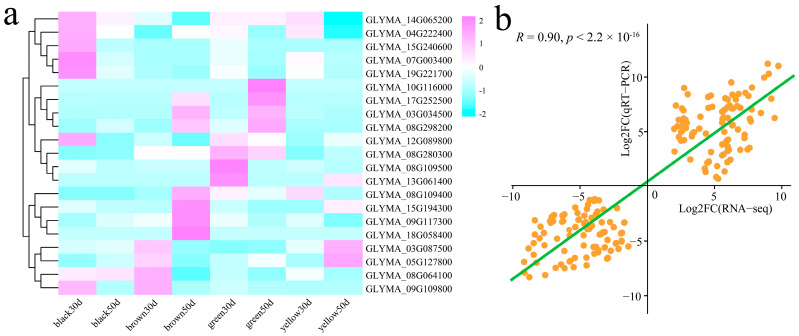
(**a**) qRT–PCR was used to generate a heatmap of the expression patterns of 21 candidate genes in different seed coat color lines. qRT–PCR was used to generate a heatmap of the expression patterns of 21 candidate genes in different seed coat color lines. (**b**) Scatter plot of RNA-seq and qRT–PCR correlations.

The expression patterns of these 21 candidates were detected via qRT–PCR (Figure 10a). Six genes (*GLYMA_14G065200*, *GLYMA_04G222400*, *GLYMA_15G240600*, *GLYMA_07G003400*, *GLYMA_19G221700*, and *GLYMA_12G089800*) were the most highly expressed genes at black 30 d. Four genes (*GLYMA_10G116000*, *GLYMA_17G252500*, *GLYMA_03G034500*, and *GLYMA_08G298200*) were the most highly expressed at brown 50 d and green 50 d. The expression of three genes (*GLYMA_08G280300*, *GLYMA_08G109500*, and *GLYMA_13G061400*) were greatest at green 30 d. The expression of four genes (*GLYMA_08G109400*, *GLYMA_15G194300*, *GLYMA_09G117300*, and *GLYMA_18G058400*) were the highest at brown 50 d. *GLYMA_03G087500* and *GLYMA_05G127800* presented the highest values at brown 30 d and yellow 50 d. *GLYMA_08G064100* and *GLYMA_09G109800* were the highest in the black 30 d and brown 30 d. In addition, the correlation between the qRT–PCR and RNA-seq fold changes for these 21 candidate genes involved in seed coat pigmentation was calculated. The results revealed that the RNA-seq data were significantly correlated with the qRT–PCR data (R = 0.90, *p* < 0.01), indicating that the RNA-seq data were reliable (Figure 10b).

## 4. Discussion

The soybean seed coat mainly contains anthocyanins and proanthocyanidins, and the composition ratio and distribution of the two determine the degree of color of the seed coat [33]. The green bean seed coat mainly contains chlorophyll, and the chlorophyll content determines the degree of green color [34]. The gene that controls the green seed coat exists in wild soybeans and is an ancient gene, but the wild soybean seed coat has a relatively high content of anthocyanins, making the seed coat appear black. After the soybeans were domesticated, yellow seed coats were used mainly for food; black beans were included in the list of traditional Chinese medicines. With improvements in living standards and increased health awareness, black beans and green beans have gradually become popular in the market [35]. Seed coat color and cotyledon color are closely related to appearance quality and intrinsic nutritional value. In this study, DMACA dyeing experiments were conducted on soybean seed coats with four colors. The surface color of the seed coats began to change approximately 46 days after seeding (Figure 2). The green color of the seed coat of soybeans with a brown seed coat deepened 46 days after seeding and lightened 48 days after seeding. This may be because the soybeans are in the middle and late stages of maturity, and a large amount of water evaporates, causing the green color of the seed coat to deepen. However, as the seeds mature further, the color of the seed coat gradually becomes lighter and finally becomes light brown. The soybeans with brown seed coats appear to have a light blue color at the hilum 46 days after seeding. The color gradually deepened and finally became dark blue at 48 days after seeding. This may be because soybeans with brown seed coats start to appear 46 days after seeding. Proanthocyanidins begin to be produced and are dyed blue by the dye. The black seed coat of soybeans begins to become dyed deeper purple 46 days after seeding and gradually turns black as the seeds mature, finally turning purple-black. This finding shows that black seed coat soybeans produce high levels of proanthocyanidins 46 days after seeding, which are quickly converted into anthocyanins under high-temperature conditions. Anthocyanins are purple under acidic conditions and are produced in large quantities as seeds mature [36]. The green 50 d and brown 30 d samples presented similar staining results, and the DEGs also presented similar expression patterns in these two periods, indicating that the green seed coat may be the key gene regulating seed coat color. It began to be highly expressed at 50 d, but the seeds were close to maturity at this time and could not change their seed coat colors faster. We found that the black seed coat and brown seed coat did not present similar expression patterns according to the DEGs and differentially expressed TFs, which showed that although the timing of anthocyanin synthesis was close, the genes and mechanisms controlling the colors of these two different seed coats were different. Moreover, the genes related to anthocyanin synthesis were expressed at the highest level in the black seed coat, which also revealed that the formation of the brown seed coat was related to the anthocyanin content, but the relationship was not very strong.

Anthocyanins play an important role in the color formation of plant seed coats [37]. The color of plants, including flowers, seed coats, and fruits, is also closely related to the anthocyanin pathway; thus, studying the anthocyanin pathway has become a popular research topic [37]. In recent years, many studies have explored the role of the flavonoid pathway in plant color, including flowers, seed coats, and fruit color. Studies have shown that key enzymes in the anthocyanin synthesis pathway, such as phenol aminotransferase, hydroxyphenylacetyl cofactor, and hydroxyphenylacetone reductase, play important regulatory roles in the development of plant color [38,39]. In addition, some studies have shown that the signal transduction pathway in the anthocyanin pathway also has an important effect on the development of soybean seed color [40,41]. These include genes that can encode and regulate related enzymes involved in the synthesis of flavonoid-related products. These genes can encode chalcone synthase (CHS), chalcone isomerase (CHI), flavanone 3-hydroxylase (F3H), anthocyanin synthase (ANS), etc. These genes are expressed at the highest level at 50 d (Figure S2). Previous studies have shown that *GmCHS1* and *GmCHS3* can form long inverted repeats (LIRs) [42]. This region silences the expression of CHS genes in a tissue-specific manner through RNA interference (RNAi), making the seed coat yellow. We also found that two new CHS genes (*GLYMA_01G228700* and *GLYMA_11G011500*) presented the lowest expression levels in yellow seed coats and the highest expression levels in black seed coats, indicating that *GLYMA_01G228700* and *GLYMA_11G011500* can be used as important candidate genes for soybean seed coat color. R2R3-MYB (*Glyma.09G235100*) can transcriptionally regulate ANS genes to produce anthocyanins and form black seed coats [43]. However, our study revealed that the expression level of *GLYMA_01G214200* (ANS) was lower in black seed coats than in yellow seed coats, which indicates that *GLYMA_01G214200* may be a negative regulator of anthocyanin content in soybean seed coats. The discovery of these genes provides important information for the improvement of the functions of cyanidin-related genes and the regulatory mechanism of the soybean seed coat.

Sugar metabolism plays an important regulatory role in plants and affects plant growth and development, energy supply, and stress resistance; thus, its relationship with plant color cannot be ignored [44]. Research has shown that sugar metabolism can affect the synthesis and accumulation of plant pigments [45]. Carbohydrates, such as glucose and fructose, can be used as raw materials for pigment synthesis and can participate in the synthesis of pigments such as anthocyanins and carotenoids. On the other hand, sugar metabolism can also affect the synthesis and transport of plant pigments by regulating related enzyme activities and gene expression [45]. For example, high sugar stress conditions may lead to the inhibition or promotion of the pigment synthesis pathway, thereby affecting the color of plants. In addition, studies have shown that some signaling pathways and regulatory factors related to sugar metabolism can also affect plant color [46]. For example, the interaction of sugar signaling pathways and phytohormones may affect phytochrome synthesis and seed coat color formation [47]. We also found that many DEGs were significantly enriched in the starch and sucrose metabolism, glycosphingolipid biosynthesis, riboflavin metabolism, cutin, suberin, and wax biosynthesis, and glycolysis/gluconeogenesis pathways. Some of the 21 candidate genes associated with these pathways were annotated.

Light is an important energy source for plants to carry out photosynthesis, and it is also an important factor affecting the synthesis of plant pigments [48]. The synthesis and accumulation of plant anthocyanins regulated by exogenous sugars and hormones mainly rely on light signals [49]. The interaction between light and sugar can affect the metabolism of plant anthocyanins by affecting the expression of certain genes. Some studies suggest that sugars not only serve as a carbon source to participate in the synthesis of light-induced anthocyanins but also may serve as signaling molecules to participate in the expression of light-induced CHS [50]. Some studies also suggest that an increase in soluble sugars in plant tissues can promote the biosynthesis of plant anthocyanins, but this sugar-induced anthocyanin synthesis depends on light signals [47]. Studies have shown that ethylene inhibits sucrose-induced anthocyanin synthesis and accumulation under light conditions by inhibiting the expression of sucrose transport proteins [51]. The effects of cytokinin on anthocyanins act downstream of the photosynthetic electron transport chain, activating the expression of the positive regulatory factors *PAP1*, *GL3*, and *TT8* and inhibiting the transcription level of the negative regulatory factor *MYBL2*, thus participating in sucrose-induced anthocyanin biosynthesis [52]. Plants are exposed to the alternating effects of light and darkness in an environment where day and night alternate, which affects the synthesis and accumulation of plant pigments, thereby affecting the color expression of plants. In addition, the circadian rhythm can affect the accumulation and distribution of plant pigments. Some plant pigments are photosensitive, and the intensity of and periodicity of changes in light can affect the distribution and accumulation of plant pigments. For example, some plants accumulate more anthocyanins in their epidermal cells when the light is strong, making the petals more colorful [53]. We also identified several genes related to the circadian rhythm among the soybean DEGs. We will later study the mechanism by which these genes influence soybean seed coat color to better understand the soybean seed coat color formation process and provide a more theoretical basis for the control and improvement of plant color.

We screened 21 candidate genes through WGCNA, including six TFs (*GLYMA_08G280300* (AP2/ERF), *GLYMA_07G003400* (bHLH), *GLYMA_19G221700* (WRKY), GLYMA_03G087500 (MYB), *GLYMA_09G109800* (MADS), and *GLYMA_08G298200* (MYB)), and three genes of the flavonoid pathway (*GLYMA_08G109500* (CHS), *GLYMA_08G109400* (CHS), and *GLYMA_04G222400* (CHI)). The expression of plant anthocyanin biosynthetic genes is regulated by various transcription factors. The main substance that controls anthocyanin accumulation in tomatoes is the MBW ternary complex, namely MYB-bHLH-WD40, in which the MYB transcription factor plays a major role, and also in cabbage [54,55]. In the dicotyledonous plant *Arabidopsis thaliana*, both MYB and HY5 can regulate related anthocyanin synthesis-related genes [55]. Transcriptome analysis of black and pink seed coat peanut varieties revealed that the MBW transcription complex regulates the anthocyanin metabolism pathway at the transcriptional level. Among them, the two MYB transcription factors with the most significantly upregulated expression in black seed coat peanuts are R2R3-MYB (which plays a key role), which is related to anthocyanin synthesis, and the HY5 protein, whose expression is upregulated in black peanuts [21]. *LhWRKY44* activates the expression of *LhF3H* and *LhGST* by directly binding to the W-box on the *LhGST* promoter, thereby promoting the accumulation of lily anthocyanins [56]. By clustering the differentially expressed TFs, we found that some TFs, including the MYB, AP2/ERF, and bHLH TFs, were highly expressed specifically at 50 d in brown. These TFs are essential for the formation of brown seed coats. The ectopic expression of *GmMYBA2* in soybean hairy roots can activate known genetic loci related to anthocyanin synthesis and increase the accumulation of delphinium-type and cyanidin-type anthocyanins in the W1t and w1T backgrounds, respectively [57]. The color of the seed coat of *GmMYBA2*-OE transgenic soybean plants in the W1 background was phenotypically similar to that of the incomplete black soybean seed coat (W1/w1, i, R, t). *GmMYBA2* can interact with the bHLH transcription factor *GmTT8a*, directly activating the expression of anthocyanin synthesis-related genes in soybeans. *GmMYBA2* and *GmMYBR* form a feedback loop to regulate the formation of soybean seed coat color. *GmTT8a* and *GmMYBR* activated by *GmMYBA2* increase and hinder the formation of the *GmMYBA2*-*GmTT8a* transcriptional activation complex, respectively [57].

## 5. Conclusions

In this study, we performed transcriptome sequencing on the seed coats of four soybean lines with different colored seed coats (medium yellow 14, black, green, and brown) 30 and 50 days after seeding. We identified several important regulatory pathways involved in soybean seed coat color by identifying genes and transcription factors that are differentially expressed between and within lines. In addition, we screened 21 candidate genes related to soybean seed coat color through WGCNA, including six transcription factor genes and three flavonoid pathway genes. However, the exact role of these genes in determining soybean seed coat color remains to be determined. Our findings provide a theoretical basis for a deeper understanding of the molecular mechanisms underlying differences in soybean seed coat color and provide new genetic resources.

## Figures and Tables

**Table 1:** Statistics of the annotated transcripts in the eight databases.

Database	COG	GO	KEGG	KOG	Pfam	Swissprot	TrEMBL	Nr	All
Number	17,860	42,447	11,047	28,613	43,237	42,272	55,817	56,835	56,848

**Table 2:** Functional annotation of 21 candidate genes.

Gene Id	Gene Name	Functional Annotation
GLYMA_08G109500	CHS	Flavonoid biosynthetic process
GLYMA_08G280300	AP2/ERF	Seed development
GLYMA_03G034500	NB-ARC	Defense response
GLYMA_03G087500	MYB	Anthocyanin synthesis
GLYMA_04G222400	CHI	Flavonoid biosynthetic process
GLYMA_05G127800	Probable starch synthase 4	Starch biosynthetic process
GLYMA_07G003400	bHLH	L-serine biosynthetic process
GLYMA_08G064100	L-galactose dehydrogenase	Amino acid metabolic process
GLYMA_08G109400	CHS	Flavonoid biosynthetic process
GLYMA_08G298200	MYB	Cell cycle
GLYMA_09G109800	MADS	Floral organ development
GLYMA_09G117300	SDR	Oxidoreductase activity
GLYMA_10G116000	Cytochrome P450	Secondary metabolites biosynthesis
GLYMA_12G089800	Calmodulin-like	Calcium ion binding
GLYMA_13G061400	UDP-glycosyltransferase	Flavonoid biosynthetic process
GLYMA_14G065200	Serine/threonine-protein phosphatase	Serine/threonine phosphatase activity
GLYMA_15G194300	Photosystem I reaction center subunit psaK	Photosynthesis
GLYMA_15G240600	F3′H	Flavonoid biosynthetic process
GLYMA_17G252500	Glucose-1-phosphate	Starch biosynthetic process
GLYMA_18G058400	lipid phosphate phosphatase	Phospholipid metabolic process
GLYMA_19G221700	WRKY	Fruit development

## Data Availability

The RNA-seq data presented in the study are deposited in the NCBI repository under accession number PRJNA1173953.

## Supplementary Materials

The following supporting information can be downloaded at https://www.mdpi.com/article/10.3390/genes16010044/s1: Table S1: All primers used in this study; Table S2: Summary statistics for RNA-seq analysis of soybean; Table S3. Expression levels (FPKM) of all DEGs; Table S4: Gene coexpression network of all modules; Figure S1: Beinong 108, green pea and its progeny green, black, and brown soybean seed coat phenotypes; Figure S2: Expression heatmap of key genes in the anthocyanin pathway, scaling the expression level to FPKM by row (−2 to 2).
