# Supplementary figures and images for: Identifying Candidate Genes Related to Soybean (Glycine max) Seed Coat Color via RNA-Seq and Coexpression Network Analysis
Source: Genes (Basel). 2025 Jan 1;16(1):44. doi: 10.3390/genes16010044 (PMC11764550; doi:10.3390/genes16010044)

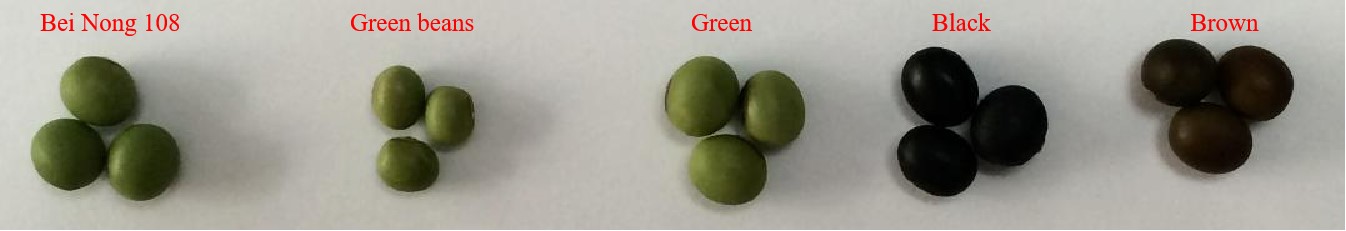

Supplement: Supplementary file 1 [file genes-16-00044-s001.zip › Fig S1.jpg]

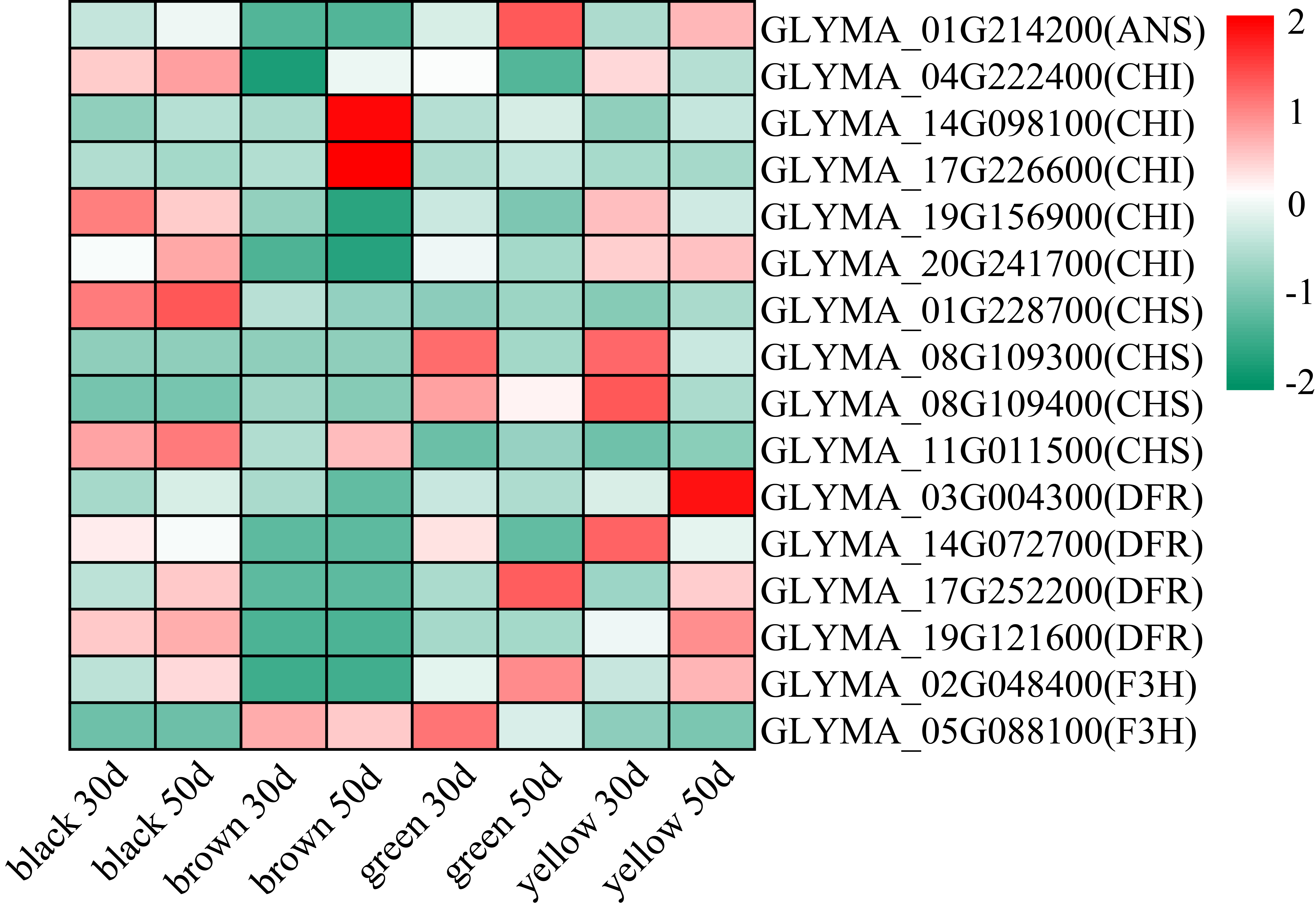

Supplement: Supplementary file 1 [file genes-16-00044-s001.zip › Fig S2.jpg]
